# Supplementary figures and images for: Metagenomics reveals novel microbial signatures of farm exposures in house dust
Source: Front Microbiol. 2023 Jun 21;14:1202194. doi: 10.3389/fmicb.2023.1202194 (PMC10321240; doi:10.3389/fmicb.2023.1202194)

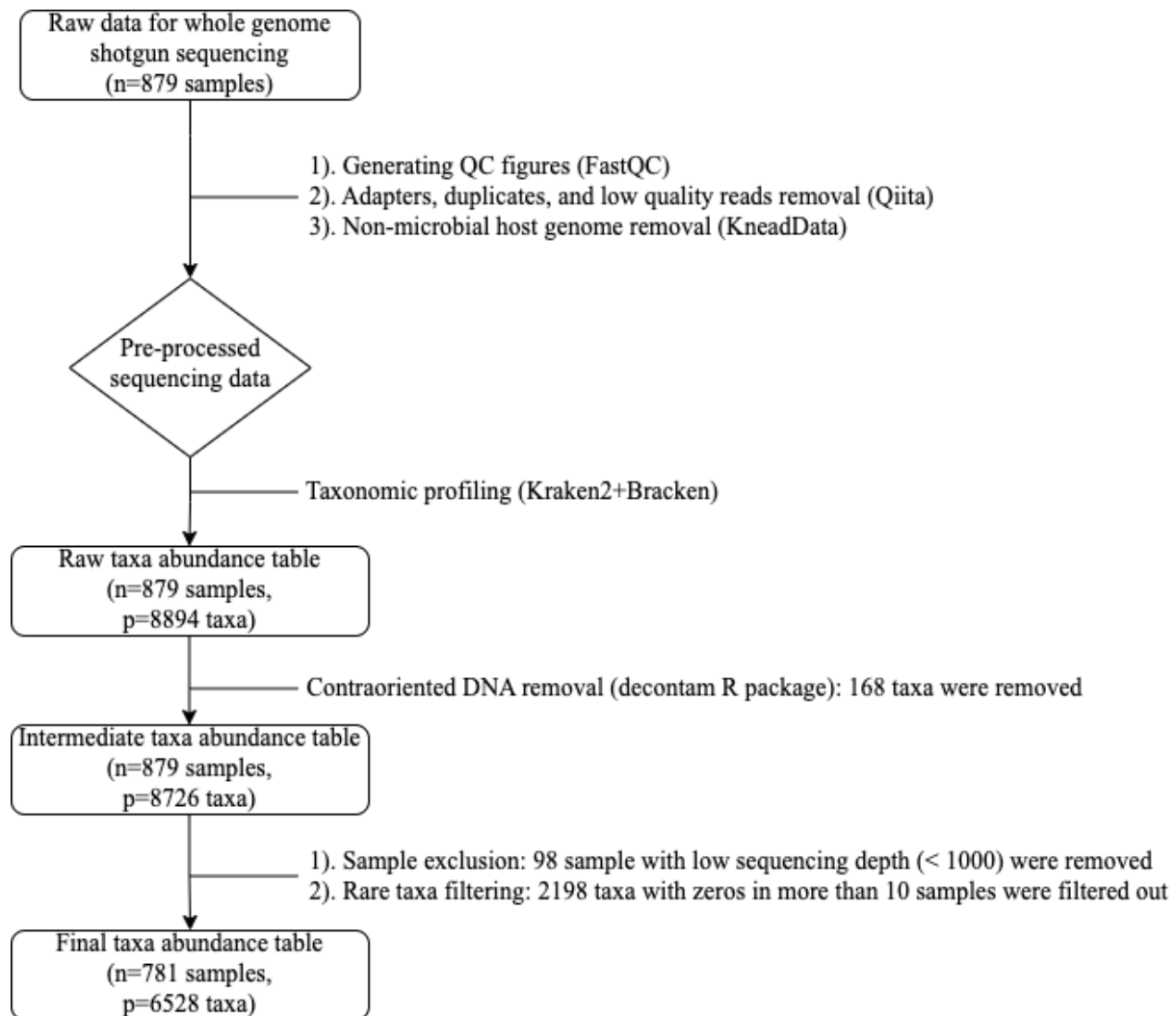

Supplementary Figure S1. Workflow of the Quality Control for WGS.

Supplement: Supplementary file 2 [file Image_1.pdf]
