# Supplementary material for: Metagenomics reveals novel microbial signatures of farm exposures in house dust
Source: Front Microbiol. 2023 Jun 21;14:1202194. doi: 10.3389/fmicb.2023.1202194 (PMC10321240; doi:10.3389/fmicb.2023.1202194)
Supplement: Supplementary file 3 [file Image_2.pdf]

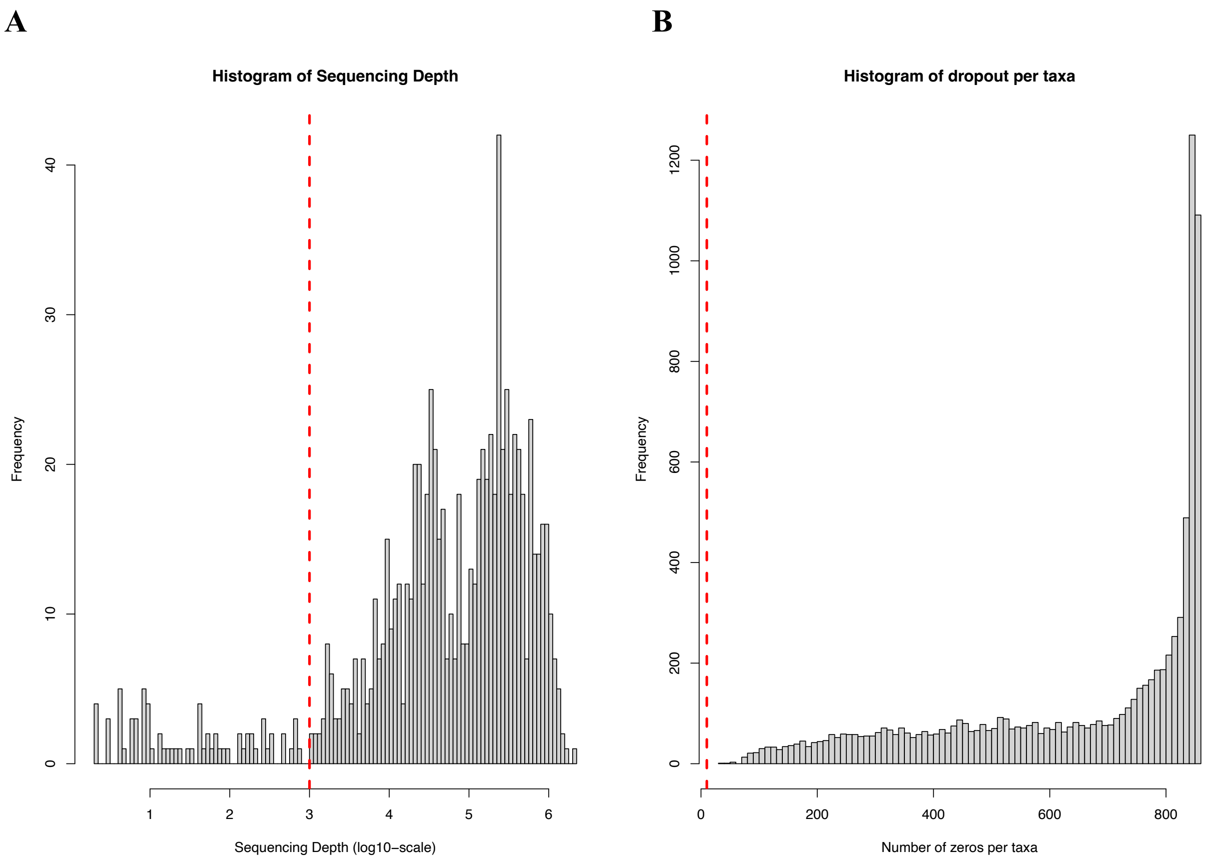

Supplementary Figure S2. Sample and rare taxa filtering criteria for WGS. **(A).** Histogram of sequencing depth. **(B).** Histogram of number of zeros per taxon. Red dotted lines indicate the threshold.
