# Supplementary material for: Metagenomics reveals novel microbial signatures of farm exposures in house dust
Source: Front Microbiol. 2023 Jun 21;14:1202194. doi: 10.3389/fmicb.2023.1202194 (PMC10321240; doi:10.3389/fmicb.2023.1202194)
Supplement: Supplementary file 5 [file Image_4.pdf]

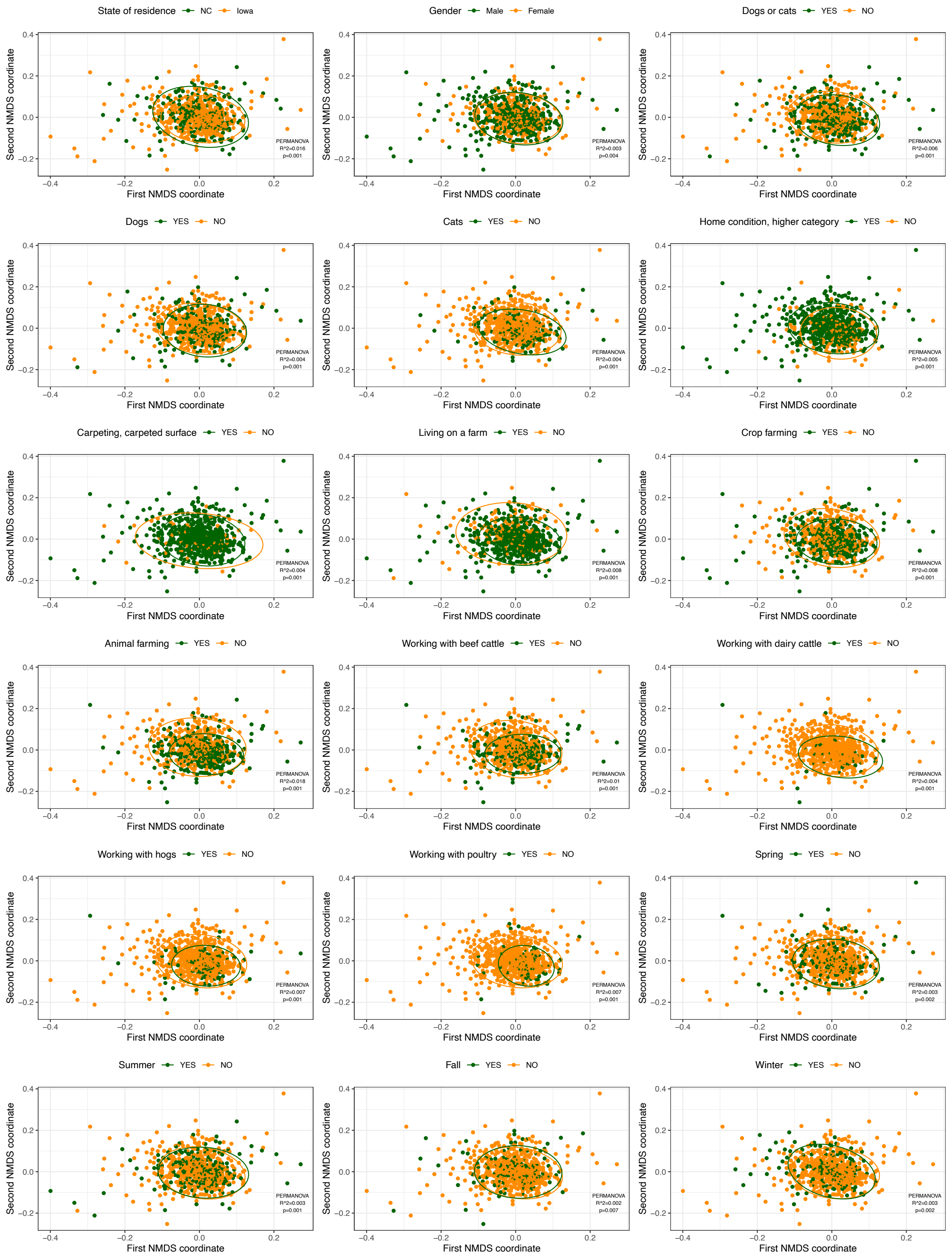

Supplementary Figure S4. Non-metric multidimensional scaling (NMDS) analysis based on weighted UniFrac distances for all exposures. The dust microbial community of each sample is indicated with one dot. R^2 value (percentage of variance explained by exposure) and p-value from the PERMANOVA analysis are reported.
